# Hematologic Features of Alpha Thalassemia Carriers

**Published:** 2012

**Authors:** Haleh Akhavan-Niaki, Reza Youssefi Kamangari, Ali Banihashemi, Vahid Kholghi Oskooei, Mandana Azizi, Ahmad Tamaddoni, Sadegh Sedaghat, Mohsen Vakili, Hassan Mahmoudi Nesheli, Soraya Shabani

**Affiliations:** 1 *Cellular and Molecular Biology Research Center (CMBRC), Babol University of Medical Sciences, Babol, Iran. *; 2 *Genetic Laboratory of Amirkola Children Hospital, Babol University of Medical Sciences, Babol, Iran.*; 3 *Non-Communicable Pediatric Diseases Research Center, Babol University of Medical Sciences, Babol, Iran.*; 4 *Ayatollah Roohani Hospital, Babol University of Medical Sciences, Babol, Iran.*

**Keywords:** Alpha thalassemia, mean cell volume, mean cell hemoglobin, mutation

## Abstract

Alpha thalassemia (α-thal) is relatively common worldwide. Most carriers are defective in either one or two alpha globin genes out of four functional ones, with deletions being more common than point mutations. The hematologic features are very important for the selection of the appropriate molecular tests while determining the genotype. The aim of this study was to compare hematologic features of patients with various types of α globin mutations. Hematological indices including red blood cells (RBC), hemoglobin concentration (Hb), mean cell volume (MCV), mean cell hemoglobin (MCH), Mean corpuscular hemoglobin concentration (MCHC) and percentage of Hemoglobin (HBA_1, _HBA_2_ and HBF) of seven-hundred and twenty two patients presenting ten different α-thal genotypes were considered. All patients showed reduced MCV and/or MCH values.Moreover, MCV and MCH were lower in patients with two functional alpha globin genes in comparison to patients with one mutated alpha globin gene (P value<0.001). In conclusion, MCV and MCH valuescan be helpful for the selection of the appropriate molecular tests to determine the genotype of alphathalassemia carriers.

Alpha thalassemia (α-thal) is one of the hemoglobinopathy that is characterized by a quantitative reduction of the α globin chains (1-2). α-thal is most common in Southeast Asia but is also prevalent in the Mediterranean, Middle East, India, and sub-Saharan Africa, with carrier frequencies ranging from 15% to 30% (3).

Each person have a pair of α globin genes, α1 and α2, on chromosome 16 (4). There are two main classes of α thalassaemia. α^0^ thalassaemias in which both α globin genes of a chromosome are deleted and α^+^ thalassaemias in which only one of the α globin genes is lost or inactive. In this later case α^+^ thalassaemia is also represented as (α^T^α/αα) (5). Individuals with only one globin deletion(-α/αα), are silent carriers and asymptomatic. Dysfunction of two α globin genes (-α/-α or --/αα ) produces mild anemia, while deletion/mutation of three α globin genes (-α/--) causes a more severe anemia characterized by production of Hb H, infection/inflammation induced hemolysis and acute or chronic cholecystitis (6-7). Complete absence of α globin genes (--/--) results in Hemoglobin Bart’s hydrops fetalis which is characterized by severe intrauterine anemia resulting in fetal hydrops and, in almost all cases, intrauterine death (8). α-thal trait diagnosis is based on microcytosis (MCV < 80 fL, MCH < 27 pg) and normal hemoglobin HB A2 level (<3.5%) (9).

α-thal most frequently results from deletion of one (-α) or both (--) α genes from the chromosome (10). 3.7 kb deletion (-α^3.7^) and 4.2 kb deletion (-α^4.2^) are the most common causes of α^+^ thalassaemia and Mediterranean deletion (--^Med^), South East Asia deletion(--^SEA^) are frequent causes of α^0^ thalassaemia (11). Also point mutations in critical regions of the alpha globin genes can cause disease, so-called, non-deletional α thalassaemia including: polyadenylation site mutations polyA1(AATAAA > AATAAG), polyA2 (AATAAA > AATGAA) and IVS-I donor site [GAG GTG AGG>GAG G-----](-5 nt) and termination codon mutations, Hb Constant Spring (10, 12). 

The accurate characterization of the hematologic features is very important for the selection of the appropriate molecular tests to determine the carrier genotype. The basic hematological tests usually used, include: the measurement of the mean corpuscular volume (MCV), the mean corpuscular haemoglobin (MCH) value and the quantity of Hb A_2_ and Hb F (13). Wide α-thal alleles have been identified inαglobin genes however limited studies were performed for considering their interaction and possible genotype-phenotype correlation. In this study, we compared hematologic features and Hb profiles of Iranian patients with various types of α globin mutations. 

## Materials and methods

Seven-hundred and twenty two adult α-thal carriers (376 males and 346 females) presenting with mild anemia were included in the study. They originated from northern provinces of Iran. Patients had been screened for α globin mutations using Gap-PCR, Reverse dot blot or restriction enzyme digestion (14,15). Of them, 215 patients had one point mutation (polyA1:14, polyA2:133, -5 nt:48, Hb Constant Spring:32), 446 patients had one deletion mutation ((-α^3.7^: 367, -α^4.7^: 54, --^Med^ : 62), 61 patients had two mutations (-α^3.7^/-α^3.7^: 50, -α^3.7^/-α^4.2^: 6, -α^3.7^ /α^ PA2^α: 7, -α^3.7^/ α^CSP^α: 5).

Patients were analyzed for hematological indices, including red blood cells (RBC), hemoglobin concentration (Hb), mean cell volume (MCV), mean cell hemoglobin (MCH), Mean corpuscular hemoglobin concentration (MCHC) and percentage of HB A_1 _and HB A_2_; and HB F (for some cases). The data were analyzed using the SPSS 16 software. Descriptive statistics including mean and standard deviation were used to describe hematologic indices of each thalassemia genotype.

## Results

Ten different α-thal genotypes in Seven-hundred and twenty two patients were compared based on hematological indices. Hematologic data of both male and female patients were considered together because there were no significant differences in hematologic indices between the 2 sexes (Tables 1 and 2) with exception of RBC and HB level that were higher in males (Tables 3 and 4). 

**Fig 1:**
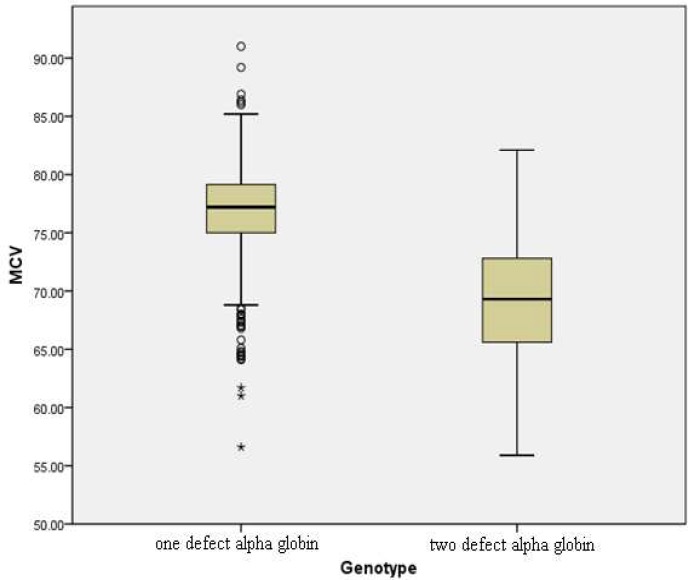
Variations (mean ± SD) of MCV values in α-thal carriers

**Fig2:**
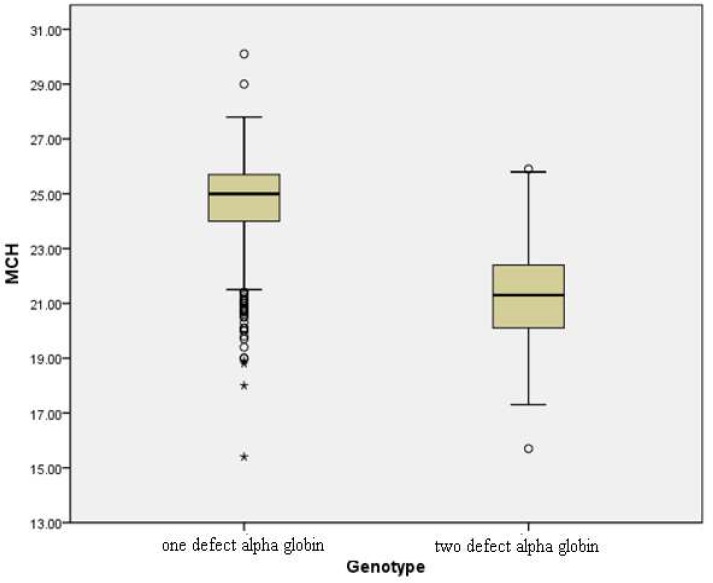
Variations(mean ± SD) of MCH values in α-thal carriers

Patients who had one mutated alpha globin gene, showed reduced MCV and MCH values. Moreover, MCV and MCH were lower in patients with two functional alpha globin genes in comparison to patients with one mutated alpha globin gene (P value<0.001). Figures 1 and 2 show the variations of MCV and MCH values respectively for the studied subjects. Although MCHC values were slightly lower in patients presenting one mutation compared to those having two functional alpha globin genes, but this difference was not significant (Tables 1and 2). Other hematological indices such as HbA2 and HbF levels as well as RBC and total Hblevel in either sexes showed no significant differences between patients presenting one and those presenting two alpha globin gene defects.

**Table 1:** Variation (mean ± SD) in Hematologic Features of α-thal carriers with one defected alpha globin gene

	MCV (fl)	MCH (pg)	MCHC ( g/dl)	A_1_(%)	A_2_ (%)	HBF (%)
α^PA1^α /αα	74.86±4.15	23.35±1.10	31.34±0.79	96.86±0.36	2.22±0.66	0.67±0.12
α^PA2^α /αα	76.81±3.32	24.65±1.47	31.96±1.38	96.87±0.61	2.49±0.44	0.71±0.27
α^5NT^α /αα	76.42±4.60	24.14±1.33	31.93±1.12	96.83±0.69	2.54±0.38	0.73±0.45
α^CSP^α /αα	75.62±4.14	24.20±1.67	32.47±1.18	96.84±0.58	2.77±0.45	0.63±0.29
-α^3.7^/ αα	76.82±5.02	24.70±1.60	32.11±1.21	96.68±2.75	2.53±0.40	0.70±1.00
-α^4.2^/ αα	77.01±3.96	24.58±2.16	31.86±1.64	96.82±0.52	2.40±0.35	0.85±0.39

**Table 2:** Variation (mean ± SD) in Hematologic Features of α-thal carriers with two defected alpha globin genes

	MCV(fl)	MCH(pg)	MCHC(g/dl)		A_1_(%)	A_2_(%)	HBF(%)
--^MED^ /αα	66.48±4.62	20.21±1.37	30.66±1.52	96. 28±0.55		2.44±0.46	0.74±0.26
-α^3.7^/ -α^3.7^	72.76±4.55	22.46±1.73	31.32±1.35	96.82±0.59		2.44±0.44	0.69±0.25
-α^3.7^/ -α^4.2^	70.71±5.17	22.43±1.85	31.41±1.20	97.20±0.70		2.25±0.53	0.50±0.17
-α^3.7^ /α^ PA2^α	70.33±2.89	21.91±0.68	30.95±1.13	97.16±0.56		2.56±0.47	0.90±0.20
-α^3.7^/ α^CSP^α	69.35±3.10	22.00±1.54	31.56±1.22	97.33±1.44		1.85±0.97	0.50±0.20

**Table 3:** Variation (mean ± SD) in red blood cells (RBC) counts and Hemoglobin (Hb) levels of α-thal carriers with one defected alpa globin gene

		n	RBC(×10^12^/L)	HB(g/dl)
α^PA1^α /αα	Male Female	5 8	6.25±0.29 5.29±0.40	14.60±0.50 12.33±0.82
α^PA2^α /αα	Male Female	70 55	5.77±0.43 5.09±0.42	14.30±1.01 12.39±1.14
α^5NT^α /αα	Male Female	19 24	5.86±0.56 5.09±0.54	14. 27±1.10 12.30±1.31
α^CSP^α /αα	Male Female	14 14	5.62±0.37 5.03±0.35	14.15±0.84 12.93±2.80	
-α^3.7^/ αα	Male Female	179 155	5.83±0.47 5.10±0.42	14.42±1.19 12.49±1.00	
-α^4.2^/ αα	Male Female	25 26	5.82±0.50 5.13±0.32	14.08±2.34 12.55±0.84	

**Table 4:** Variation (mean ± SD) in red blood cells (RBC) counts and Hemoglobin (Hb) levels of α-thal carriers with two defected alpa globin genes

		n	RBC(×10^12^/L)	HB(g/dl)
--^MED^ /αα	Male Female	24 30	6.22±0.83 5.49±0.48	12.62±1.81 11.13±.89
-α^3.7^/ -α^3.7^	Male Female	27 18	5.97±0.45 5.37±0.518	13.55±1.28 11.98±1.03
-α^3.7^/ -α^4.2 ^	Male Female	1 5	5.29 5.28±0.47	13.40 11.46±0.55
-α^3.7^ /α^ PA2^α	Male Female	4 2	6.28±0.26 5.62±0.02	13.63±0.32 12.05±0.21
-α^3.7^/ α^CSP^α	Male Female	3 1	5.75±0.59 5.08	12.73±0.63 10.80

## Discussion

Alpha Thalassemia is a serious disease for many of Mediterranean, Middle East and Southeast Asian countries (3). It is estimated that in Iran nearly 25% of the annual blood production is used for thalassemic patients. Moreover, progresses in treatment of disease and increase in the life expectancy of thalassemic patients is expected to increase the prevalence of thalassemic population (16). Premarital screening programs for identification of carrier of thalassemia and prenatal diagnosis of disease for couples at risk of having affected child, is an effective way for control of thalassemia (17) that was carried out in some countries like Iran (18-19).

The key to identifying the globin genes mutations in carriers and affected patients is an understanding of the genotype/ phenotype relationships of the various globin gene mutations and the effects of interaction when several mutations are co-inherited.

Consideration of hematological indices of α-thal genotypes indicated that all thalassemia genotypes may not be associated with mild hematological phenotypes (Tables 1 and 2). Although there were statistically significant differences in MCV, and MCH between genotypes, it was difficult to distinguish the different conditions based on hematological data. However comparison of MCV and MCH between patients with two functional alpha globin genes and patients who had one defected alpha globin gene, showed noticeable decreases (P value<0.001). Therefore MCV and MCH can be useful for prediction and distinction of the genotype of α-thal patients. Limited phenotype/genotype studies of α-thal patients showed similar results (20-21). 

Although hematologic features of thalassemic newborns did not show similar reduction of MCV and MCH, but there was similar difference in MCV and MCH of subjects with two functional alpha globin genes and patients who had one defected alpha globin gene (22). We found no significant differences between genotypes and other hematological indices such as Hb level, MCHC and RBC counts in this study.

In conclusion, analysis of MCV and MCH can be efficiently helpful for the selection of the appropriate molecular tests to determine the genotype of alpha thalassemia carriers. 
